# Supplementary material for: Juvenile and Osteoarthritic Human Chondrocytes Under Cyclic Tensile Strain: Transcriptional, Metabolic and Kinase Responses
Source: Int J Mol Sci. 2025 Nov 12;26(22):10934. doi: 10.3390/ijms262210934 (PMC12652661; doi:10.3390/ijms262210934)
Supplement: Supplementary file 1 [file ijms-26-10934-s001.zip › ijms-3906202-supplementary.pdf]

Supplementary data

Juvenile and osteoarthritic human chondrocytes under cyclic tensile strain: transcriptional, metabolic, and kinase responses

Birgit Lohberger[1,2*], Vincent Grote[3], Heike Kaltenegger[1,2], Dietmar Glänzer[1,2], Patrick Sadoghi[1], Tanja Kraus[1], Bibiane Steinecker-Frohnwieser[2*]

[1] Department of Orthopedics and Traumatology, Medical University Graz, Graz, Austria

[2] Ludwig Boltzmann Institute for Arthritis and Rehabilitation, Saalfelden, Austria

[3] Ludwig Boltzmann Institute for Rehabilitation Research, Vienna, Austria

***corresponding authors**: ResProf. PD Dr. Birgit Lohberger, MSc; Department of Orthopedics and Traumatology, Medical University Graz; Auenbruggerplatz 5, A-8036 Graz; email: birgit.lohberger@medunigraz.at

PD Dr. Bibiane Steinecker-Frohnwieser; Ludwig Boltzmann Institute for Arthritis and Rehabilitation, Department for Rehabilitation, Thorerstrasse 26, A-5760 Saalfelden; email: Bibiane.Steinecker-Frohnwieser@lbg.ac.at

Table S1. Intercorrelations [r] of respected BIOMs for jCH in a) and pCH-OA in b) under baseline conditions are given. Values represent Pearson correlation coefficients between individual BIOMs, indicating the strength and direction of linear relationships. All data were collected under standardized baseline conditions. Significant correlations ($p < 0.05$) are indicated in bold.

BIOMs	ACAN	ADAMTS4	ADAMTS5	BGLAP	BMP2	cEBP	COL1	COLX	IL6	IL8	MMP1	MMP13	MMP3	RUNX2	SOX5	SOX6	SOX9	SPP	TLR4
**ACAN**		-0.25	-0.05	0.26	**0.47**	-0.27	**0.46**	-0.10	0.00	**-0.50**	**0.42**	**0.45**	0.18	-0.23	-0.03	0.30	**0.68**	**-0.60**	-0.10
**ADAMTS4**			0.04	-0.37	**-0.72**	**-0.50**	0.07	-0.05	-0.37	**-0.50**	**0.52**	-0.09	-0.16	**0.56**	**-0.59**	**-0.53**	**-0.61**	0.07	**-0.38**
**ADAMTS5**				**0.57**	0.35	-0.17	0.21	**0.78**	**0.82**	-0.27	**-0.39**	**0.68**	0.22	-0.23	-0.03	**0.57**	-0.36	0.04	-0.17
**BGLAP**					**0.60**	-0.22	**0.59**	**0.81**	**0.69**	0.04	**-0.59**	0.22	0.38	-0.26	0.27	**0.76**	0.24	0.04	0.35
**BMP2**						0.26	0.32	0.28	**0.54**	0.05	**-0.39**	0.38	**0.53**	**-0.70**	**0.53**	**0.81**	**0.64**	**-0.52**	0.06
**cEBP**							**-0.66**	**-0.44**	0.21	**0.65**	-0.13	-0.13	-0.18	**-0.40**	0.25	-0.16	0.02	-0.13	0.35
**COL1**								**0.50**	0.01	**-0.53**	-0.09	-0.01	**0.44**	0.20	-0.08	**0.51**	**0.39**	-0.23	-0.04
**COLX**									**0.64**	-0.11	**-0.66**	0.27	0.23	0.00	0.07	**0.65**	-0.17	0.31	0.13
**IL6**										0.15	**-0.53**	**0.60**	0.21	**-0.46**	0.21	**0.59**	-0.19	0.11	0.15
**IL8**											**-0.55**	**-0.46**	-0.07	-0.30	**0.52**	-0.05	0.02	**0.39**	**0.54**
**MMP1**												0.10	-0.35	0.32	**-0.61**	**-0.65**	-0.03	**-0.43**	-0.27
**MMP13**													0.18	**-0.43**	0.00	**0.42**	-0.06	-0.26	**-0.46**
**MMP3**														**-0.61**	**0.71**	**0.72**	**0.42**	-0.29	**-0.52**
**RUNX2**															**-0.80**	**-0.59**	**-0.44**	**0.39**	0.23
**SOX5**																**0.60**	**0.49**	-0.05	-0.16
**SOX6**																	**0.46**	-0.21	-0.15
**SOX9**																		**-0.55**	0.06
**SPP**																			0.26
**TLR4**																			

Table S1. a) Intercorrelations [r] of BIOMs for jCH under baseline conditions.

BIOMs	ACAN	ADAMTS4	ADAMTS5	BGLAP	BMP2	cEBP	COL1	COLX	IL6	IL8	MMP1	MMP13	MMP3	RUNX2	SOX5	SOX6	SOX9	SPP	TLR4
**ACAN**		-0.07	0.23	**0.61**	**0.58**	**-0.59**	0.07	**0.48**	-0.30	0.21	**0.69**	**0.73**	**0.78**	-0.19	**0.44**	0.17	0.06	-0.35	**-0.68**
**ADAMTS4**			-0.20	**0.57**	-0.04	-0.27	0.34	0.05	-0.36	**-0.71**	**0.43**	0.22	0.11	**0.84**	**-0.43**	**-0.48**	0.19	-0.18	-0.08
**ADAMTS5**				-0.07	0.35	0.10	**0.59**	0.10	**0.72**	-0.26	0.06	0.25	**0.42**	-0.35	**0.61**	**0.72**	-0.24	**0.43**	0.22
**BGLAP**					**0.47**	**-0.53**	0.17	0.25	**-0.47**	-0.14	**0.71**	**0.66**	**0.74**	**0.53**	-0.07	**-0.38**	0.24	-0.25	**-0.47**
**BMP2**						**-0.70**	-0.23	-0.04	-0.05	0.31	0.37	**0.80**	**0.83**	-0.22	**0.51**	-0.01	**-0.46**	-0.15	**-0.40**
**cEBP**							0.38	-0.02	**0.61**	-0.20	**-0.42**	**-0.72**	**-0.55**	0.10	-0.10	0.29	0.37	**0.61**	**0.82**
**COL1**								**0.51**	**0.53**	**-0.81**	0.26	-0.02	0.15	0.37	0.25	**0.46**	**0.47**	**0.40**	0.34
**COLX**									0.13	-0.30	**0.41**	0.19	0.24	0.15	**0.56**	**0.42**	**0.73**	-0.05	-0.26
**IL6**										-0.25	-0.31	-0.24	-0.06	-0.22	**0.57**	**0.71**	0.04	**0.69**	**0.69**
**IL8**											-0.16	0.11	0.11	**-0.62**	0.08	-0.07	-0.31	-0.17	-0.31
**MMP1**												**0.82**	**0.73**	0.31	0.10	0.07	0.23	-0.12	**-0.43**
**MMP13**													**0.91**	-0.03	0.33	0.10	-0.17	-0.17	**-0.57**
**MMP3**														-0.01	**0.44**	0.15	-0.07	-0.06	**-0.44**
**RUNX2**															**-0.42**	**-0.55**	**0.47**	-0.01	0.21
**Sox5**																**0.65**	0.01	0.17	-0.04
**SOX6**																	0.13	**0.46**	0.12
**Sox9**																		0.08	0.00
**SPP**																			**0.65**
**TLR4**																			

Table S1. b) Intercorrelations of BIOMs for OA under baseline conditions.

Table S2. The table presents data on the effect of mechanical stimulation on jCH and pCH-OA. The effect between unstimulated control cells and mechanically stimulated cells is represented as fold changes in mean values and standard deviations are given. Statistical significance was evaluated using the Student's t-test and is indicated as follows *: $p < 0.05$; **: $p < 0.01$; ***: $p < 0.001$.

BIOM	group	*n*	mean value	SD	t-test [p]
**MMP1**	jCH	9	1.81	0.52	0.002 **
	pCH-OA	9	1.27	0.35	<0.05 *
**MMP3**	jCH	9	1.29	0.59	0.003**
	pCH-OA	9	1.12	0.34	0.35
**MMP13**	jCH	9	2.48	2.08	0.09
	pCH-OA	9	1.56	0.81	0.15
**ADAMTS4**	jCH	9	1.40	0.27	0.013*
	pCH-OA	9	1.69	0.44	0.02*
**ADAMTS5**	jCH	9	1.44	0.27	0.001 **
	pCH-OA	9	1.53	0.26	0.002 **
**COL1A1**	jCH	9	1.02	0.39	0.86
	pCH-OA	9	1.17	0.48	0.77
**COLX**	jCH	9	0.84	0.14	0.009 **
	pCH-OA	9	0.80	0.27	0.84
**ACAN**	jCH	9	1.60	0.39	0.023*
	pCH-OA	9	1.65	0.58	0.02 *
**BMP2**	jCH	9	1.51	0.35	0.002 **
	pCH-OA	9	1.75	0.21	<0.001 ***
**RUNX2**	jCH	9	1.16	0.25	0.023*
	pCH-OA	9	0.93	0.22	0.06
**SPP1**	jCH	9	3.03	0.48	<0.001 ***
	pCH-OA	9	3.46	1.60	0.002 **
**BGLAP**	jCH	9	1.06	0.50	0.65
	pCH-OA	9	1.45	0.28	0.34
**SOX5**	jCH	9	1.44	0.35	0.01 *
	pCH-OA	9	1.46	0.73	0.09
**SOX6**	jCH	9	0.97	0.29	0.75
	pCH-OA	9	0.91	0.31	0.93
**SOX9**	jCH	9	1.24	0.18	0.01*
	pCH-OA	9	1.05	0.26	0.28
**IL6**	jCH	9	0.91	0.25	0.67
	pCH-OA	9	0.76	0.16	0.002 **
**IL8**	jCH	9	1.17	0.69	0.03 *
	pCH-OA	9	0.59	0.39	0.008 **
**TLR4**	jCH	9	1.78	0.74	0.02*
	pCH-OA	9	1.05	0.50	0.45
**cEBP**	jCH	9	1.08	0.19	0.24
	pCH-OA	9	0.95	0.27	0.92

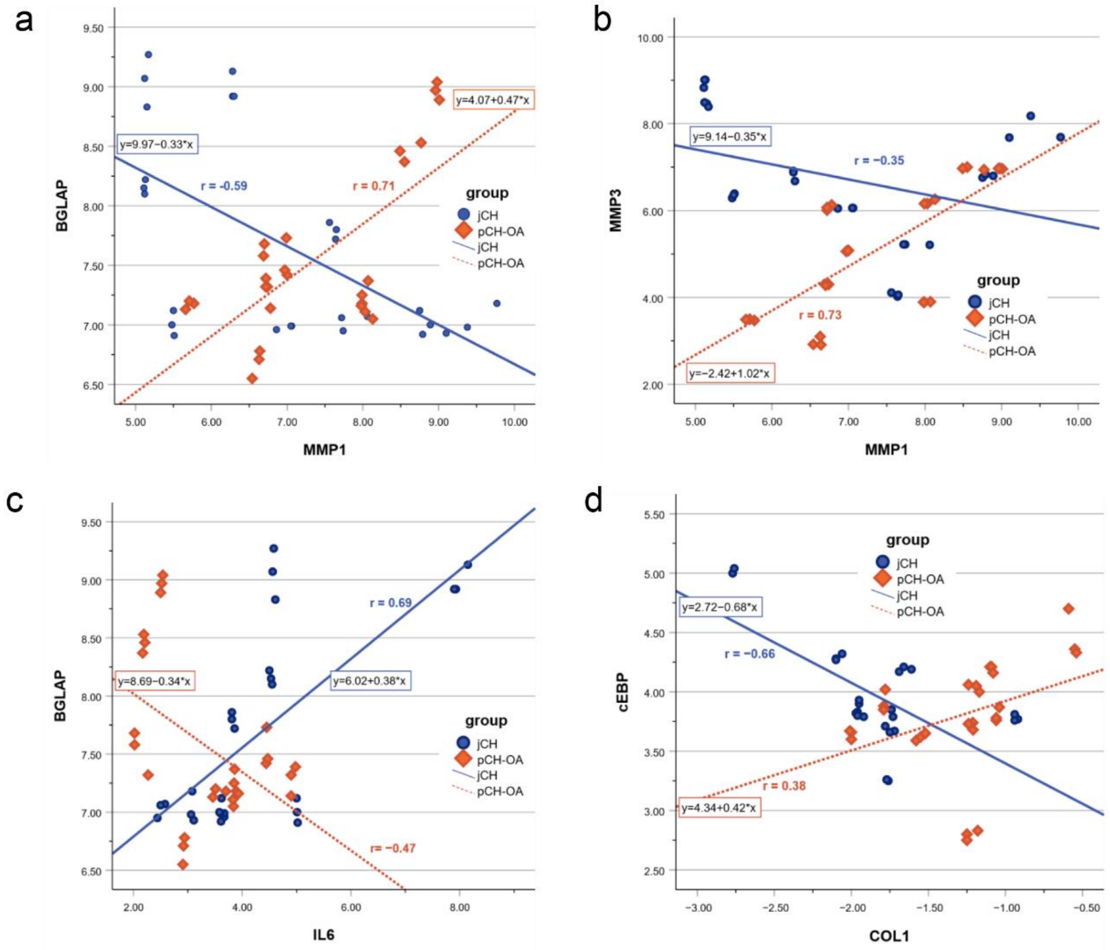

Figure S1. Scatter plots a - d depicting differences in intercorrelations of specific BIOMs between jCH (blue) and pCH-OA (red). Each scatter plot shows the Pearson correlation coefficients ([r]) of BIOMs for both cell types, highlighting the strength and direction of relationships between them. In addition, the linear regression equations are given for each pair of variables. The data illustrate variations in intercorrelations between the two cell types, providing insights into the functional dynamics of the BIOMs. Statistical significance of the differences is assessed and indicated, with values of $p < 0.05$ considered significant.
